# Review: Neuroprotective Nanocarriers in Glaucoma

**DOI:** 10.3390/ph17091190

**Published:** 2024-09-10

**Authors:** Kun Pei, Maria Georgi, Daniel Hill, Chun Fung Jeffrey Lam, Wei Wei, Maria Francesca Cordeiro

**Affiliations:** 1 UCL Institute of Ophthalmology, London EC1V 9EL, UK; kun.pei.21@ucl.ac.uk (K.P.); d.hill.13@ucl.ac.uk (D.H.); 2 St Mary’s Hospital, Imperial College Healthcare NHS Trust, London W2 1NY, UK; m.georgi@imperial.ac.uk; 3 Department of Surgery & Cancer, Imperial College London, London SW7 5NG, UK; w.wei20@imperial.ac.uk; 4 GKT Medical School, Guy’s Campus, Great Maze Pond, London SE1 1UL, UK; chun.lam@kcl.ac.uk; 5 Imperial College Ophthalmic Research Group (ICORG) Unit, Imperial College, London NW1 5QH, UK; 6 Western Eye Hospital, London NW1 5QH, UK

**Keywords:** glaucoma, ocular neurodegenerative disease, nanomedicine, nanocarriers, neuroprotection, neuroprotective, retinal ganglion cell

## Abstract

Glaucoma stands as a primary cause of irreversible blindness globally, characterized by the progressive dysfunction and loss of retinal ganglion cells (RGCs). While current treatments primarily focus on controlling intraocular pressure (IOP), many patients continue to experience vision loss. Therefore, the research focus has shifted to therapeutic targets aimed at preventing or delaying RGC death and optic nerve degeneration to slow or halt disease progression. Traditional ocular drug administration, such as eye drops or oral medications, face significant challenges due to the eye’s unique structural and physiological barriers, which limit effective drug delivery. Invasive methods like intravitreal injections can cause side effects such as bleeding, inflammation, and infection, making non-invasive delivery methods with high bioavailability very desirable. Nanotechnology presents a promising approach to addressing these limitations in glaucoma treatment. This review summarizes current approaches involving neuroprotective drugs combined with nanocarriers, and their impact for future use.

## 1. Introduction

Glaucoma, a principal cause of irreversible blindness globally [1,2], remains a multifactorial neurodegenerative condition with an unclear pathogenesis. It is marked by the gradual degeneration and structural damage to RGCs, primarily manifesting as progressive visual field loss [3]. Due to neural compensatory mechanisms for visual deficits, patients typically present late with gradual and often asymmetrical vision loss [4]. The current estimated global prevalence of glaucoma in a population aged 40–80 years of age is 3.54%. In 2013, it was estimated that 64.3 million people worldwide between the ages of 40 and 80 had glaucoma. This number increased to 76.0 million by 2020 and is projected to reach 111.8 million by 2040 [5]. However, these figures are likely an underestimation with over 90% of glaucoma cases in low- and middle-income countries remaining undiagnosed [6,7]. Glaucoma also poses significant direct and indirect financial loses, with an estimated annual cost of USD 2.9 billion in the United States [6].

The Glaucoma Research Foundation recognizes multiple glaucoma subtypes, including primary and secondary open-angle, primary and secondary closed-angle, and normal tension glaucoma. Risk factors include elevated intraocular pressure, genetic predisposition, myopia, ethnicity (such as Afro-Caribbean), and hypertension [7,8]. Currently, elevated IOP remains the only modifiable risk factor that medical intervention can target [9,10]. Most current treatments therefore try to lower IOP through surgery, laser, or medication to prevent or delay the progression of RGC degeneration. 

**Figure 1:**
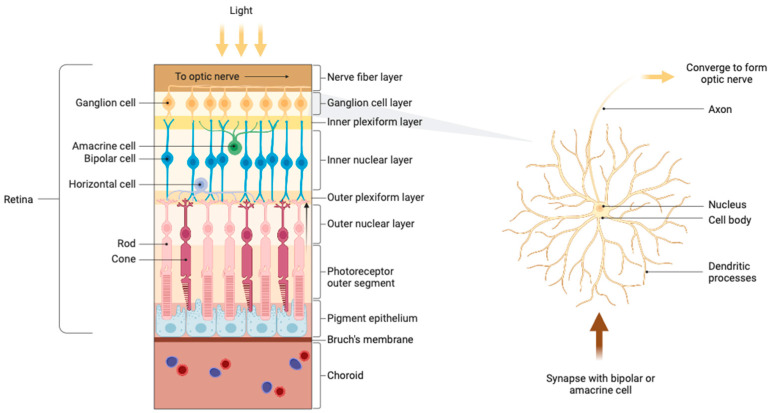
Anatomy of the retina and ganglion cells.

Although most current treatments have resulted in effectively controlled IOP, these approaches are not optimal as they fail to address the persistent loss of RGCs [11]. RGCs serve a pivotal function in visual processing by transmitting information from the retina to the brain and are the primary cellular component of the ganglion cell layer (GCL) [11,12]. The adult human eye contains approximately 1.2 million RGCs [13]. Cell bodies, intricate dendritic structures, and distinctive axons make up the structural components of RGCs [11]. Dendrites process photoreceptor impulses and receive synaptic input mostly from amacrine and bipolar cells, which act as intermediary neurons in the retina to transmit and modulate information from the photoreceptors (Figure 1). These interactions form a critical hierarchical network essential for the integration and processing of visual signals into nerve impulses. The action potentials generated then travel via the axons of RGCs, which merge to form the optic nerve. These axons pass through the optic disc and optic canal, forming synapses in the lateral geniculate nucleus (LGN) of the thalamus and partially in the superior colliculus (SC), thereby linking the visual pathway of the eye with that of the brain [14]. The LGN processes visual information, relaying it to the primary visual cortex for further complex analysis and integration, highlighting the crucial role of RGCs in visual perception [13,15].

Mechanisms leading to RGC apoptosis in glaucoma include ischemia [16], oxidative stress [17,18], mitochondrial dysfunction [17], diminished levels of neurotrophic factor [18,19], accumulation of misfolded proteins [20], neuroinflammation [21], and mechanical stress due to elevated intraocular pressure [22,23]. Despite concerted efforts by ophthalmologists to use pharmacological, laser, and surgical methods to lower intraocular pressure, these strategies are not always successful in protecting retinal ganglion cells [24]. Furthermore, the side effects associated with these treatments present significant concerns, particularly adverse reactions to the medications or complications from surgical procedures [25]. This underscores the complexity of treating glaucoma and highlights the need for developing more effective and safer therapeutic approaches. The neuroprotective approach aims to preserve RGCs and their axons independently of IOP, lowering the number of interventions [26]. This approach has gained prominence as it seeks to directly address the cellular and molecular pathways involved in RGC damage, providing a potentially more comprehensive treatment paradigm for glaucoma [26]. Neuroprotection focuses on protecting neurons from predominantly secondary neurodegeneration to improve health outcomes [27,28]. The primary target of neuroprotection in glaucoma is the retinal ganglion cell. RGC loss is irreversible [15]; thus, the protection of these cells is essential. Neuroprotection in glaucoma provides a supplementary therapy for those whose progressive visual loss is not sufficiently mitigated by IOP reduction alone [25,27]. In addition, it has potential to serve as an alternative and complementary therapy for patients who are intolerant or unresponsive to IOP-lowering treatments, thus broadening the scope of potential treatment options in glaucoma management [25,27].

Numerous studies have investigated neuroprotective therapies for glaucoma, identifying several promising avenues. These include neurotrophic factors [29], dietary supplements [27,30,31], anti-inflammatory therapies [32,33], α2-adrenoceptor agonists [34], N-methyl-D-aspartate (NMDA) receptor antagonists [35], calcium channel blockers [36], and metabolic-targeting compounds [37]. Table 1 provides a summary of the contemporary neuroprotective treatments for glaucoma. These therapies, which are either undergoing preclinical evaluation or are currently being tested in clinic, have demonstrated promising results in terms of efficacy. Each therapeutic avenue targets a unique pathway, offering diverse mechanisms to mitigate the neurodegenerative processes in glaucoma. The diversity of neuroprotective therapeutic strategies reflects the evolving understanding of the complex pathophysiology of glaucoma, with each pathway offering a unique potential therapeutic target.

Among the various challenges faced by neuroprotective therapies, a significant barrier to their effectiveness is the blood–retinal barrier (BRB), which plays a crucial role in the eye’s posterior segment, tightly regulating and isolating the retina’s microenvironment from systemic circulation [38]. The BRB is composed of two main components: the internal BRB (iBRB) and the external BRB (oBRB) [38]. The iBRB is formed by tight junctions between endothelial cells of the retinal capillaries, which originate from the central retinal artery. It serves the inner retinal layers through three vascular plexuses, effectively controlling the internal environment. The oBRB, on the other hand, includes tight junctions between retinal pigment epithelial cells, the choroid, BM, and the RPE [38]. The choroid’s capillaries are crucial for supplying oxygen and nutrients to the outer retinal layers, removing waste products, and maintaining retinal temperature and volume [39]. The BM, located between these capillaries and the RPE, allows for selective molecular diffusion and blocks larger molecules, which helps stabilize the RPE and absorb physical stress [40]. These barriers prevent water, plasma, and toxic compounds from penetrating the eye. They may also impede the administration of therapeutic drugs [38].

**Figure 2:**
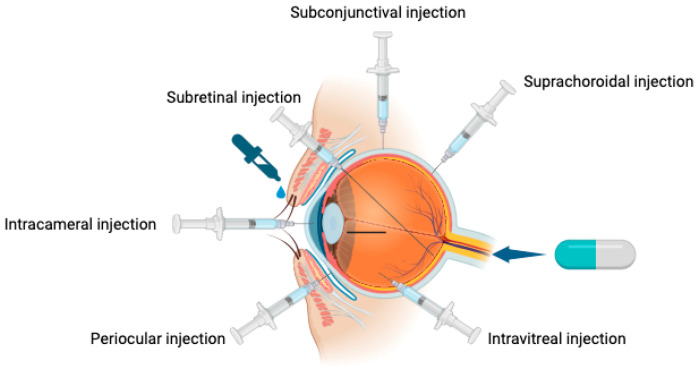
Ocular drug delivery systems.

The effectiveness of common treatment methods is significantly compromised by the barrier effect of the BRB. Currently, the most prevalent methods of ocular drug delivery include topical administration or intracameral, suprachoroidal, subconjunctival, intravitreal, and subretinal injections, and finally, systemic administration (Figure 2) [41]. Table 2 presents the advantages and disadvantages of these modes of administration. These methods achieve varying degrees of therapeutic success, especially as their bioavailability is generally low, typically less than 5% (largely due to the BRB [42]). As a result, it is important to develop innovative techniques overcoming or bypassing the challenges imposed by the BRB. 

**Figure 3:**
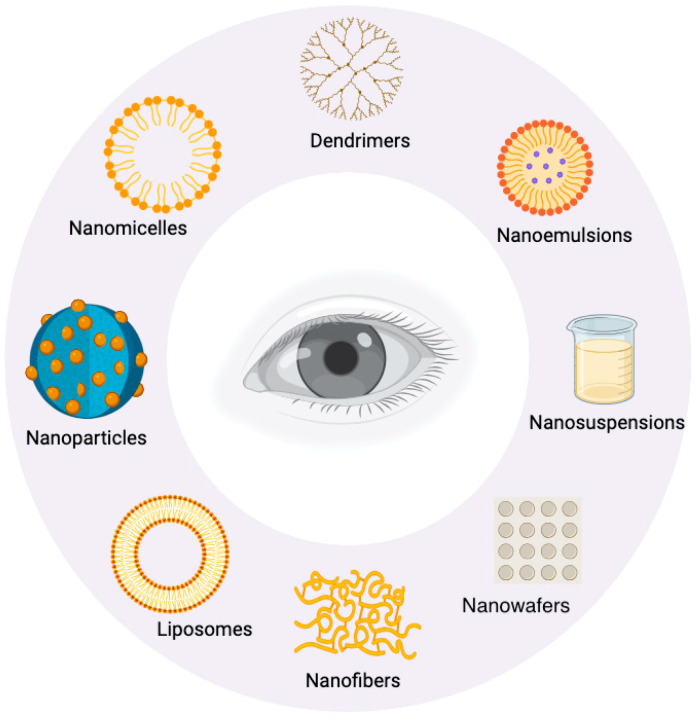
Nanocarrier formulations for ocular drug delivery.

The BRB’s effects on drug bioavailability limit the beneficial effects of neuroprotective agents using conventional drug delivery methods. However, advances in nanotechnology have significantly pushed the field of ocular drug delivery forward [43]. Nanocarriers including micelles, nanoemulsions, solid lipid nanoparticles, dendrimers, liposomes, and exosomes have shown promise compared to the established methods of drug delivery [44]. The design of these novel carriers aims to overcome ocular barriers, improve drug stability, and boost therapeutic efficacy [44]. They also extend the half-life of the drugs in ocular tissues, and reduce the frequency of dosing; these factors improve patient compliance and decrease drug-related side effects [45]. Furthermore, nanocarriers enable sustained and controlled release, targeted drug delivery, and the combination of multiple drugs, resulting in improved treatment outcomes for a variety of ocular conditions [45] (Figure 3).

Nanocarriers 1.2 exhibit a range of mechanisms of action which enhance the delivery and efficacy of neuroprotective agents. 

Dendrimers are symmetrical synthetic polymers which consist of three architectural domains: a central core, a hyperbranched mantle, and a corona with peripheral reactive functional groups (dendrons). Their unique properties, including hyperbranching and high biocompatibility with biological systems, make them valuable in a wide range of medical and biomedical applications [46]. These three-dimensional nanopolymeric structures are particularly promising as vectors for drug and gene delivery. Enhanced drug delivery is achieved by increasing the solubility of hydrophobic drugs through the entrapment of neuroprotective agents within the intramolecular cavities of dendrimers. Additionally, the exterior functional groups allow for the attachment of targeting moieties, improving the specificity of drug delivery to certain diseases [47]. However, dendrimers can interact with biological membranes, potentially resulting in apoptosis and hemolysis. In vivo studies have demonstrated hepatotoxicity and renal toxicity of higher-generation dendrimers [48]; further in vivo investigations into the surface modification and safety characterization of dendrimers are required. 

Liposomes are spherical phospholipid vesicles with a cell-like membrane which provides low immunogenicity, low toxicity, and high biocompatibility [49]. They can encapsulate either hydrophobic or hydrophilic agents, protecting drugs from hydrolysis and prolonging their biological half-life, while controlling drug release [50]. Liposomes are limited by the low entrapment efficiency of water-soluble molecules, rapid hemofiltration and excretions, and fast drug release (Gireesh, T., C. Kuldeep, and K. Pramod. “Liposomal current status, evaluation and recent advances” [51]). 

Nanoparticles (NPs) are drug carriers ranging from 1 to 1000 nm in size. They can be divided into polymer and lipid nanoparticles [52]. Ocular NPs are primarily composed of lipids, proteins, and either natural or synthetic polymers [53]. Nanoparticles (NPs) can be classified into nanocapsules and nanospheres based on their morphological structure. Nanospheres possess a solid polymeric structure, while nanocapsules feature a thin polymeric shell, about 5 nm thick, encasing an oily core. These NPs have the capability to encapsulate both hydrophobic and hydrophilic drugs, protecting them from degradation and enhancing targeted delivery. This results in more efficient drug absorption, controlled release, and improved bioavailability [54]. 

Their absorption efficiency is significantly affected by their surface charge [55]; catatonic NPs, for instance, exhibit higher retention times on ocular surfaces when compared to anionic NPs due to the negatively charged surfaces on corneal and conjunctival tissues [55]. NPs have several advantages, specifically regarding ocular drug delivery; (1) their small size reduces ocular irritation; (2) their superior absorption abilities enable the sustained drug release and therefore reduce dosing intervals; (3) they feature improved cellular penetration due to their better absorption; (4) they enable targeted drug delivery to desired tissues [56,57,58].

Although NPs hold significant potential for the management of ocular diseases, severe challenges hinder their widespread use in clinical practice. These challenges include insufficient drug loading, premature drug release during storage, difficulties in achieving uniform particle dispersion, and toxic effects associated with surfactant concentrations [59]. Further research is needed to advance the clinical application of NPs.

Table 3 summarizes recent research focused on neuroprotective drugs combined with nanocarriers, specifically for the protection of RGCs in glaucoma treatment. This review categorizes and discusses these studies, as well as addresses the safety of nanocarriers and their impact on therapeutic efficacy. The existing literature explores the effects of neuroprotective drugs combined with nanocarriers in retinal diseases, as well as the use of neuroprotective drugs in treating glaucomatous conditions. This review comprehensively summarizes the current impact of neuroprotective drugs combined with nanocarriers, specifically on glaucoma.

**Table 1:** Neuroprotective drugs for anti-glaucoma or retinal ganglion cell protection.

Neuroprotectant Class	Drug (Mode of Delivery)	Mechanism
α2-adrenoceptor agonists	Brimonidine [60] (topical, SC)	It decreases the production of aqueous humor and increases uveoscleral outflow, meaning it is commonly used to manage glaucoma due to these hypotensive properties. Alpha2-adrenoceptors have been found in the retinal ganglion layer and inner nuclear layer, indicating potential neuroprotective advantages. They can upregulate retinal expression of anti-apoptotic proteins, including bcl-2 and bcl-x, thereby protecting the RGCs.
N-methyl-D-aspartate (NMDA) receptor antagonists	Memantine [61] (oral)	A non-competitive NMDA receptor antagonist that specifically targets activated glutamatergic receptors, reducing toxic calcium influx and excessive glutamatergic activity, while preserving normal neurotransmission. It has demonstrated protective effects on RGCs in rat models.
Calcium channel blockers	Brovincamine [62] (topical) Flunarizine [63] Nilvadipine [64]	Prevent calcium-mediated apoptosis and increase ocular blood flow by inhibiting calcium influx into vascular smooth muscle cells. This leads to peripheral vasodilation, reduced vascular resistance, and increased blood flow to the optic nerve.
Metabolic-targeting compounds	Metformin [65] (oral)	Reduces oxidative stress by targeting fibrotic signaling, oxidation of nicotinamide adenine dinucleotide (NAD), and mitochondrial energetics.
	Insulin (SC) [66]	A peptide hormone with receptors distributed throughout the central nervous system, including the retina, which operates through the PI3K/Akt signaling pathway to protect retinal neurons, glial cells, and vasculature from excessive glucose flux and apoptosis.
Neurotrophins	NGF [67] (topical, IVT)	Neurotrophins are transported to the retina in a retrograde manner and regulate neuronal growth, function, and survival. Elevated IOP in glaucomatous conditions obstructs this retrograde transport. NGF is expressed in both the retina and target brain cells. In the retina, NGF binds to Tyrosine kinase receptor A (TrkA) on RGCs to promote neural differentiation and prevent apoptosis.
	BDNF [68]	BDNF, through its receptor TrkB, demonstrates strong neuroprotective effects by reducing dendritic degeneration in mouse models.
Dietary supplements	Nicotinamide (oral) [69]	A dietary supplement and redox reaction coenzyme which increases extracellular NAD+ levels, thereby increasing mitochondrial NAD+ concentration. Animal glaucoma models have demonstrated a declining capacity to retain NAD during early disease.
	Citicoline [70] (oral, IM, topical)	A structural and functional precursor for essential components cell membranes, which also reduces oxidative stress in the central nervous system through the promotion of glutathione production.
	Phosphoserine [71]	A structural matrix of cellular membranes which plays a fundamental role in the synthesis of neurotransmitters.
Anti-inflammatory therapies	Anti-complement: ANX005 [72] (IV) ANX007 [73] (IVT)	A recombinant antibody humanized against complement C1q. C1q, along with C1s2 and C1r2, constitutes the C1 complex, which helps initiate the complement pathway. In glaucoma animal models, C1q is upregulated in the retina. It contributes to the formation of the membrane attack complex, which phagocytoses weakened synapses and RGCs.
	Anti-fas–ONL1204 [74] (IVT)	Fas ligand (fragment apoptosis stimulator), a member of the TNF family, exhibits distinct properties in ocular inflammation. Soluble FasL does not trigger inflammation in the eye, whereas membrane-bound FasL induces potent inflammation. Additionally, soluble FasL inhibits corneal inflammation, which is exacerbated by the pro-inflammatory effects of membrane-bound FasL in mice models.
	MicroRNA-124 [75]	Regulates target genes expression through post-transcriptional regulation.

**Table 2:** Advantages and disadvantages of ocular drug deliveries.

Method of Delivery	Advantages	Disadvantages
Topical eye drops [76]	Non-invasive.	Ocular surface side effects. Low bioavailability especially for posterior segment tissues.
Subconjunctival injections [27]	Bypasses ocular surface.	Invasive. Limited for anterior segment pathologies.
Intracameral injections [27]	Commonly for delivering antibiotics post-operatively, especially cataract surgery, to reduce endophthalmitis rates.	Invasive. Risk of toxic anterior segment syndrome, toxic endothelial cell destruction syndrome.
Intra-vitreal injections [76]	Higher bioavailability. Relatively less technically demanding compared to other delivery methods of posterior segment pathologies.	Invasive. Risk of endophthalmitis, hemorrhage, retinal detachment.
Subretinal injections [27]	Higher bioavailability. Targeted treatment for RPE and outer retina, especially useful in gene therapy.	Invasive, requires vitrectomy. Technically challenging. Effects confined to site of injection with limited distribution.
Suprachoroidal injections [77]	Higher bioavailability. Targeted treatment for choroid, RPE, outer retina.	Technically challenging.
Systemic [27]	Non-invasive for oral administration.	Higher risk of systemic side effects. Low bioavailability.

**Table 3:** Neuroprotective nanocarrier in glaucoma studies.

Neuroprotective Strategy	Year	Nanocarrier	Neuroprotective Agent	Administration	Size	PDI	Zeta	EE	Subject	Assesment	Outcome
Antioxident	2024	GelCA, a gelatin-modified hydrogel (Cur@PDA NPs)	Curcumin	Intravitreal	328.6 nm	less than 0.3	−26.7 ± 1.1 mV	70.5 ± 10%	ONC ICR mice, RGC-5 (H_2_O_2_ induced)	Vitro: AmplexTM Red reagent (10-acetyl-3,7-dihydroxyphenoxazine), Vivo: H&E staining (Bio-Compatibility), tissue staining (Antioxidative Performance)	Cur@PDA@GelCA shows strong biocompatibility, protects retinal tissue from oxidative stress, and offers lasting adhesion.
Antioxident, anti-inflammation	2023	chitosan-hyaluronic acid (CS/HA)	EPOβ	Topical	330 ± 15 nm	0.174 ± 0.016	+28 ± 1 mV	38.4 ± 0.3%	Wistar Hannover Rat (cauterizing three episcleral veins)	ERG, IOP, Histologic Evaluation	The nanoparticle reaches the retina through topical use, significantly improving ERG and retinal thickness earlier in treated animals.
Antioxident	2023	hydroxyl PAMAM dendrimers	N-acetylcysteine (NAC) (D-NAC)	intravitreal, intravenous	5.8 nm	NA	+6.5 mV	NA	Wistar rat model of laser-induced, Translimbal laser (TLL)	IOP, Immunostaining, transcriptomic studies were performed on RNA isolated from retina	Hydroxyl PAMAM dendrimers target activated microglia/macrophages quickly post-injury and remain for 28 days, while NAC conjugation offers neuroprotection.
Antioxident, anti-inflammation	2022	chitosan-hyaluronic acid nanoparticles (CS/HA)	epoetin beta (EPOβ)	Subconjunctival	289 ± 3 nm	0.126 ± 0.085	39 ± 1 mV	38.4 ± 0.3%	Wistar Hannover rat	IOP, ERG and microhematocrit evaluations; histological evaluation (immunofluorescence and HE)	CS/HA nanoparticles enhance mucoadhesion and retention on the ocular surface, safely delivering EPOβ to the retina.
Antioxident	2018	Pluronic-F127 stabilised TPGS	Curcumin	Topical	17.9 nm	0.002	+18.69 mV	94.20%	R28 cell line, DA rat (OHT, pONT)	IOP, Immunostaining	Boosts the drug’s solubility by nearly 400,000 times, significantly preserving RGC density.
a2-adrenergic agonist	2022	Polydopamine (PDA)	brimonidine	Intravitreal	223.9 ± 4.7 nm	NA	−28.5 ± 0.58 mV	20%	vitro: Human umbilical vein endothelial cells (HUVECs), Raw 264.7, N2a, 661W and ARPE-19; vivo: C57BL/6 mice (ONC)	Nissl staining, Propidium iodide (PI) uptake and cell death analysis, RT-PCR, transcriptome analysis, Immunostaining, vivo: Optomotor Test; Light/Dark Transition Test	PDA nanoparticles effectively eliminate reactive species and reduce cellular ROS. Brimonidine-loaded PDA (Br@PDA) offers superior protection against RGC loss and visual impairment.
a2-adrenergic agonist	2016	Alkoxylphenacyl-based polycarbonates	Brimonidine tartrate (BRT)	Intravitreal	189.9–199.8 nm	NA	around −0.2 mV	less than 20%	human trabecular meshwork (HTM) cell, Wistar rats	Immunostaining, cytotoxicity studies	AP-PCL microfilms sustained BRT release for over 90 days due to slow degradation and microporous formation, showing good biocompatibility in cell studies and rat models.
a2-adrenergic agonist, IOP lowering	2015	nanosponge (NS)	Brimonidine, Travoprost, Bimatoprost	Intravitreal, Topical	Bimatoprost: 400 nm and 700 nm; Brimonidine, Travoprost: 50 nm	NA	NA	NA	C57BL/6 (C57) mice	IOP, qualification of Neuro-DiO	A single NS injection delivers ocular hypotensive drugs continuously for up to 32 days and may effectively target RGCs.
a2-adrenergic agonist	2015	human serum albumin nanoparticle (HSA-NP)	Brimonidine	Intravitreal	152.8 ± 51.1 nm	NA	−29.7 ± 7.5 mV	NA	RGC-5 cells; Sprague–Dawley (SD) rat (ONC)	Immunostaining	Br-loaded HSA-NPs provide a prolonged therapeutic effect and enhanced neuroprotection synergistically.
Neurotrophic factors	2023	Cell adhesion peptide (CAP)-gemini surfactants (18-7N(p1-5)-18)	BDNF	Intravitreal	180–320 nm	0.2–0.5	+53.7 ± 1.15 or 13.1 ± 0.5 mV	NA	CD1 mice, Rat A7 astrocyte, 3D retinal neurosphere model from CD 1–4 multipotent retinal stem cells (MRSC)	Vitro: Flow cytometry (Transfection efficiency and viability studies), Transfection study, Vivo: stained with Syto™ 13 nucleic acid stain observed CLSM, BDNF-ELISA (for BDNF expression)	IgSF CAPs were successfully used to improve the adhesion and delivery of gemini NPXs to retinal cells through conjugation with gemini surfactant gene vectors.
Neurotrophic factors	2016	K2® nanoparticle gene delivery system (K2-NPs)	BDNF	NA	83.9 ± 0.4 nm	0.17 ± 0.01	+57.3 ± 2.8 mV	NA	two-layer contact-independent 3D neuronal co-culture model	flow cytometry, and enzyme-linked immunosorbent assay (ELISA)	Quantifying neurite growth in astrocyte-SH-SY5Y cell co-cultures serves as an effective bioassay model for evaluating non-viral gene delivery systems.
NMDA receptor antagonists	2018	memantine-loaded PLGA-PEG nanoparticles (MEM-NP)	Memantine	Topical	141.8 nm	0.078 ± 0.018	−26.5 mV	80.60%	retinoblastoma (Y-79) and keratinocytes (HaCaT) cells. Dark Agouti rat (OHT). New Zealand rabbits. (DA) rats	IOP, Immunostaining, Corneal and scleral permeation	In vitro epithelial and neuronal cell cultures, this formulation was better tolerated than free memantine and effectively preserved RGC density.
blockade of glutamate transmission	2022	PLGA nanoparticles in situ gelling system (NPs-Gel)	Riluzole	Topical	below 200 nm	below 0.2	−30.0 mV	94%	C57BL6 mice	Immunostaining	Optimized PLGA nanoparticles penetrate the blood-retinal barrier, delivering RLZ for 24 h, while RLZ NP gels enhance retention, clear vision, and comfort for glaucoma and dry eye patients.
IOP lowering/antioxidant	2020	Soluplus	Melatonin/Agomelatine	Topical	61.78 ± 1.61 nm	0.068 ± 0.022	NA	NA	Vivo: Sprague–Dawley RAT (Methylcellulose (MCE) model)	IOP, ERG, Western Blot Analysis, Immunostaining	In the MCE model, melatonin/agomelatine significantly reduced IOP elevation more effectively than timolol or brimonidine.
IOP lowering, anti-inflammatory	2020	The diblock poly ethylene glycol-co-polysebacic acid (PSA-PEG)	brinzolamide and mi-RNA-124	Intravitreal	250–400 nm	NA	0.015–0.020 mV	90%	dutch-belted rabbits and 69 C57/BL6 mice	IOP, Immunostaining, real time PCR	The miRNA/NP-BRZ system is safe, non-toxic, and effectively reduces IOP while providing neuroprotection. The novel nanoparticles offer a slow release and accurately deliver BRZ to the target site, ensuring a long-lasting effect.

## 2. Therapeutic Agents

### 2.1. Brimonidine

Brimonidine, a selective α2-adrenergic receptor agonist, is primarily used in clinical settings to lower IOP by reducing aqueous humor production and enhancing uveoscleral outflow [78]. Additionally, the presence of α2-adrenergic receptors in the GCL and the INL is associated with brimonidine having neuroprotective actions [34]. These effects manifest indirectly through interactions with glial cells and directly on RGCs [79]. Research has shown that brimonidine can prevent RGC apoptosis by reducing excitotoxicity, which inhibits the release of neurotransmitters like glutamate and aspartate, and by elevating the concentration of neurotrophic growth factors [34,80,81]. Commonly available as topical eye drops (Alphagan^®^ P at concentrations of 0.1% and 0.15%, and Alphagan^®^ at 0.2%) [82], brimonidine’s effectiveness is limited by its low bioavailability (1–7%) and quick clearance from the eye, necessitating frequent application to achieve the desired outcomes [83]. For this reason, various studies have utilized nanocarriers with brimonidine with the objective of improving the efficacy and duration of their action (Table 4).

Luo et al. explored the use of polydopamine (PDA), a biocompatible melanin-like polymer derived from dopamine, as a nanocarrier for brimonidine in a glaucoma model [83]. Their research demonstrated that an intravitreal injection of brimonidine in PDA effectively reduced damaged RGCs and diminished microglial activation, while protecting the RPE [83]. Moreover, the PDA nanoparticles significantly enhanced axon regeneration compared to brimonidine alone [83]. Notably, the brimonidine–PDA formulation increased axon density tenfold from the lesion site compared to other treatments, indicating a synergistic effect. This formulation also more effectively increased RGC density and reduced microglial numbers compared to either component alone, likely due to PDA’s antioxidant properties [83]. Visual function assessments through optomotor response tests and light/dark conversion assays revealed that brimonidine–PDA markedly improved visual function. Thus, brimonidine encapsulated in PDA nanoparticles served as an effective neuroprotective nanotherapeutic, significantly enhancing ROS elimination and improving RGC protection.

A study by Kim et al. demonstrated an enhancement in the efficacy of brimonidine using human serum albumin (HSA) as carriers, which independently showed great improvements in RGC survival in the optic nerve crush model [3]. This neuroprotective effect of HSA on RGC survival is likely mediated by modulating the aggregation of amyloid-beta (Aβ) [84]. On day 5, the RGC survival rates were similar for HSA–NPs with and without brimonidine, at 52.6 ± 3.3% and 63.5 ± 7.1%, respectively. By day 14, these rates were 30.7 ± 11.7% for HSA–NPs alone and 38.1 ± 3.6% for HSA–NPs with brimonidine. The group receiving only brimonidine had a higher survival rate on day 5 (58.0 ± 4.2%), but this dropped to 18.6 ± 3.9% by day 14, indicating that the treatment effects lasted longer in both HSA groups [3]. Notably, the HSA with brimonidine (HSA–Br) group exhibited the best treatment efficacy at 14 days [3]. This enhanced outcome may be due to HSA prolonging the availability of brimonidine by supporting its continuous release, or possibly the initial neuroprotective effects of brimonidine synergistically enhancing the therapeutic impact of HSA, resulting in a more extended and potent neuroprotective effect than either HSA–NPs or brimonidine alone. Compared to Luo et al.’s research, Kim et al.’s study not only showed improved efficacy of nanocarriers but also highlighted their potential for extended therapeutic durations. However, unlike Luo et al.’s experiments, Kim et al.’s did not test for functionality.

In exploring advanced drug delivery systems for extended durations of action, alkoxylphenacyl-based polycarbonates copolymerized with polycaprolactone (AP–PCL) were used to develop both injectable nanoparticles and implantable microfilms [82]. The AP–PCL microfilms enabled an extended release of brimonidine tartrate (BRT), starting with an initial burst and then gradually releasing the drug consistently up to approximately 90 days. Importantly, the viability of human trabecular meshwork (HTM) cells exposed to these microfilms was not compromised, indicating no toxic effects. In a separate application, AP–PCL nanoparticles were injected intravitreally into Wistar rats. Observations up to 40 days post-injection showed no signs of retinal detachment, suggesting good ocular tolerance and biocompatibility of the nanoparticles. Additionally, a nanosponge technology encapsulating brimonidine was developed to extend the duration of drug release [85]. Smaller nanosponges (around 50 nm) proved as effective in reducing IOP as the topical application immediately after treatment or intravitreal injection, with effects lasting up to about a week. Larger nanosponges (about 500 nm) showed a 27% reduction in IOP lasting nearly 3 weeks post-injection, demonstrating their potential for long-term IOP management [85]. While nanosponges demonstrate enhanced capability for sustained IOP management, Lambert et al. did not perform a quantitative evaluation of RGC survival. However, considering brimonidine’s known protective effects on RGCs, this approach still holds promise for long-lasting RGC protection. It should be noted that neither study conducted in vivo functional testing, which would provide a more comprehensive and convincing assessment of the therapeutic benefits of long-term drug release.

### 2.2. Brain-Derived Neurotrophic Factor (BDNF)

BDNF is a protein produced by ganglion cells and inner nuclear layer cells that provides neuroprotection directly through the pro-myosin receptor kinase B (TrkB) expressed in RGCs or indirectly via TrkB on glial cells [86]. Increased IOP impairs the vital retrograde transport of BDNF, necessary for RGC survival, by inhibiting dynamin, a protein that assists in the transport of molecules like TrkB and BDNF [86]. In a rat model of glaucoma, the upregulation of the BDNF gene has shown protective effects on RGCs, suggesting its potential as an adjunct therapy alongside IOP reduction [87]. Although injections of neurotrophic factors like BDNF can inhibit or even reverse cell damage, their effects are transient and typically need multiple injection. Naked nucleic acids are highly susceptible to degradation without a delivery system and struggle to escape endosomes for successful nuclear translocation. Gene delivery vectors, essential for addressing these challenges, are divided into viral and non-viral systems. While viral vectors are effective at delivering nucleic acids, their potential to cause adverse immune reactions due to their mobility and immunogenicity is a concern [88]. While many viral systems have progressed to the clinical stage, nonviral systems are still under development [89]. The challenge of effectively and reliably delivering these therapies to the retina remains significant. The development of a non-viral, nano-based BDNF gene delivery vector system holds promise for addressing these issues by enabling sustained therapeutic delivery (Table 5).

Chen et al. developed an astrocyte and SH-SY5Y cell co-culture model to assess neurite outgrowth, thus providing a valuable bioassay for evaluating non-viral systems [88]. They employed the K2^®^ nanoparticle-based delivery system (K2-NPs) loaded with BDNF-encoding plasmids to transfect A7 astrocytes. Enhanced neurite outgrowth was consistently observed in trA7/SH-SY5Y and trA7/oxSH-SY5Y conditions compared to controls [88]. Additionally, the team synthesized dual surfactants modified with integrin-targeting RGD and neuroimmune immunoglobulin superfamily (IgSF) peptides to transfect genes into retinal cells in vitro and vivo [89]. In animal studies, a CD1 rodent model demonstrated that intravitreal injection of 18-7N(pFASNKL)-18pNPX resulted in superior retinal cell transfection and elevated gene expression levels of tdTomato/BDNF compared to its RGD counterpart and the parental compound, with BDNF levels 3.4 times greater than the control [89]. These findings suggest that pNPX modified with integrins and IgSF CAM-binding peptides has the potential to deliver therapeutic DNA to the target site, thereby significantly increasing BDNF secretion.

Recent research suggests that gene therapy delivering both BDNF and its receptor TrkB together produces superior results compared to administering each component alone [90]. This synergistic approach enhances axonal transport and provides superior protection for RGCs compared to isolated gene therapies. The likely mechanism for these enhanced therapeutic outcomes is the prevention of receptor downregulation, ensuring sustained receptor activation and prolonged therapeutic effects. Khatib et al.’s research supports this, demonstrating that the combination of TrkB and BDNF stimulates axonal transport more effectively than administering the receptor or ligand alone, highlighting the superior efficacy of combined treatments.

In terms of other neurotrophic factors, combination with nanocarriers significantly enhances therapeutic outcomes for RGC survival in non-glaucoma models. For instance, Wang et al. linked Pituitary adenylate cyclase-activating polypeptide 38 (PACAP38) to rat RGC exosomes, markedly improving survival rates, nerve layer thickness, axonal regeneration, and visual function recovery in a rat model of traumatic optic neuropathy (TON) [91]. Another study found that proteins like BDNF and neurotrophies, when conjugated to magnetic nanoparticles (MNPs), effectively protected against RGC loss in an oxidative stress model, while equivalent doses of free proteins were ineffective [92]. These results show the potential and benefits of combining neurotrophic factors with nanocarriers to enhance therapeutic efficacy.

### 2.3. Memantine

Memantine selectively binds to activated glutamate receptors and has been shown to protect ganglion cells from glutamate toxicity by inhibiting overactive NMDA receptors, without affecting normal receptor activity [61]. This protection stems from preventing excitotoxic damage, which is often due to excessive calcium influx through NMDA receptors, leading to free radical formation—a process implicated in the pathophysiology of glaucoma [93]. However, preclinical studies reveal that memantine offers limited benefits in glaucoma models. For example, in one study, oral memantine improved early multifocal electroretinography (ERG) responses and visual evoked cortical potential (VECP) amplitudes in monkeys at 3 and 5 months following an increase in IOP but showed no benefits at 16 months [94]. While safe, the neuroprotective effects of memantine are modest, likely due to its low bioavailability [94]. To address this, Sánchez et al. developed an eye drop using memantine-loaded nanoparticles (MEM–NPs), demonstrating good efficacy and safety [95].

In comparison with the untreated control group, the number of RGCs was significantly lower in the group with health control. Treatment with MEM–NPs effectively prevented OHT-induced RGC damage, indicating that its protective mechanism does not depend on IOP, as IOP levels in the MEM–NPs and ocular hypertension (OHT) groups were similar [95]. MEM–NPs showed no toxicity at any tested concentration, caused less irritation, and provided better penetration than the free drug [95]. The detection of memantine in scleral tissue suggests that a drug aggregate may have formed within the sclera, allowing memantine to diffuse into intraocular tissues and improving the drug’s efficacy. By reducing the dosage and focusing on localized administration, the potential for systemic side effects from memantine could be lessened, ensuring that effective drug concentrations reach the targeted tissues. However, a vehicle control group, which would have clarified the effect of the carrier alone on RGC health, was lacking in this study. Additionally, the absence of a group treated with free memantine alone precludes a direct comparison that could demonstrate the potential enhancement of therapeutic efficacy by the nanoparticle carrier. These omissions are important because they limit the final assessment of whether the observed neuroprotective effects can be specifically attributed to the nanoparticle formulation rather than to the drug itself or the vehicle.

Memantine has been shown to reduce amyloid-beta peptide levels in vitro and in Alzheimer’s disease (AD) transgenic mouse models [96]. There is increasing evidence linking amyloid-beta accumulation to the pathophysiology of glaucoma and age-related macular degeneration (AMD) [97]. Since amyloid-beta is also implicated in AMD progression, therapies like brimonidine and memantine that inhibit amyloid-beta pathways may be valuable for treating AMD [97]. Memantine has also shown promising results in Alzheimer’s disease patients by reducing excitotoxic damage to neurons [98], suggesting it could be very suitable for elderly patients with multiple neurodegenerative disorders. Current evidence does not validate memantine’s effectiveness in slowing the progression of glaucoma; hence, its off-label use for this condition is not advised. Nevertheless, employing nanoparticle carriers could introduce new therapeutic opportunities [99]. This method might improve memantine’s bioavailability and effectiveness, potentially enhancing treatment outcomes for glaucoma.

### 2.4. Riluzole (RLZ)

RLZ is an FDA-approved drug that is primarily used in the treatment of amyotrophic lateral sclerosis (ALS). Its neuroprotective effects could be attributed to several factors, although the exact mechanism of action is not fully understood. These include inhibition of voltage-dependent sodium channels, which reduces sustained sodium currents, partial protection of axons in white matter from hypoxia, and facilitation of excess glutamate removal via the glutamate transporter EAAT2. RLZ has shown neuroprotective properties in neuronal axon models and has been effective in slowing RGC death in experimental glaucoma models. However, its low water solubility could cause serious adverse effects such as pancreatitis, angioedema, and hepatitis. Using nanoparticle carriers to enhance RLZ’s solubility and enable localized administration might be a valuable strategy to reduce these adverse reactions and improve its therapeutic impact. This approach could allow for the targeted delivery of RLZ directly to affected areas, potentially minimizing systemic exposure, and maximizing its neuroprotective effects.

Esteruelas and colleagues developed a nanocarrier system for RLZ using poly (lactic-co-glycolic acid) (PLGA) nanoparticles and a nanogel formulation. Initial tests showed that both the RLZ nanoparticles (RLZ–NPs) and the RLZ–NPs gel were non-irritating within the first five minutes of application, whereas free RLZ caused mild vasoconstriction and moderate irritancy. Drug distribution studies revealed that, 24 h after administration, both formulations were primarily located in the eye posterior area, with the gel formulation more prevalent in the anterior structures compared to the nanoparticles [100]. Although RLZ–NPs reached therapeutic targets faster, they were also cleared more quickly than the RLZ–NPs gel. The gel’s greater mucoadhesive properties allow it to retain the RLZ–NPs within ocular tissues for a longer period, potentially offering more consistent dosing. Additionally, the gel possesses suitable rheological and viscosity properties that prevent visual blurriness [100]. However, this study did not include efficacy tests; it only demonstrated that the drug delivery systems could reach targeted areas within the eye. There were no evaluations of therapeutic effectiveness, such as RGC survival rates or functional tests like ERG, nor comparisons to determine whether encapsulation in carriers improved therapeutic outcomes. This focus on drug delivery without assessing actual treatment efficacy marks a significant limitation in evaluating the potential benefits of these systems for treating glaucoma.

### 2.5. Curcumin

Curcumin is a polyphenol pigment produced from herb, also known as natural Yellow 3 or E100 under the European Food Additive Directive [101]. Curcumin has shown potential in mitigating pathways related to the development of common ophthalmic diseases, including oxidative stress linked to mitochondrial activity, β-amyloid aggregation, inflammation via PPAR-γ agonist activity, inhibition of astrocyte proliferation through iNOS and JAK2-STAT3 pathways, and anti-angiogenic effects via the VEGF/VEGFR/K-ras pathway [102]. Despite its relatively low toxicity and high tolerability, curcumin faces several major obstacles that limit its clinical use. Its poor bioavailability and low water solubility (approximately 11 ng/mL) pose significant challenges [101]. Although side effects are typically mild and often limited to gastrointestinal disturbances, curcumin undergoes rapid metabolism in the liver and intestines, where it is quickly converted into water-soluble metabolites (glucuronides and sulfates) that are rapidly excreted from the body. This rapid breakdown and elimination result in low bioavailability and limit its therapeutic effectiveness [102].

A novel hydrogel system was developed by partially grafting cinnamic acid onto gelatin to create a GelCA macromonomer with a high substitution degree (91%) [103]. This GelCA hydrogel is crosslinked under UV light via photoinitiated dimerization of the olefinic groups in cinnamic acid, providing π-π stacking capabilities for drug adsorption. Polydopamine (PDA) nanoparticles were synthesized using these π-π stacking and hydrogen bonding properties to effectively load curcumin, enhancing biocompatibility. Cytotoxicity studies showed no significant differences in cell viability among GelCA, PDA-GelCA, and Cur-PDA-GelCA hydrogels, nor were there signs of tissue atrophy or cell loss in retinal tissues injected with these hydrogels after two weeks, indicating good biocompatibility. In vivo experiments using an ONC model revealed that GelCA alone did not significantly affect antioxidative properties compared to the control ONC group. However, both groups showed potential in mitigating oxidative damage after ONC, with significant improvements over the control [103]. The similar antioxidative results between two groups, as indicated by DHE staining, suggest the need for more specific in vivo tests to discern differences in their therapeutic effects [103]. Despite Cur-PDA nanoparticles demonstrating superior efficacy in inhibiting DPPH• radicals and other ROS compared to PDA alone, the distinct therapeutic benefits of curcumin remain to be fully established. Given the comparable therapeutic outcomes and the invasive nature of intravitreal injections, further investigations related to drug release duration and minimizing injection frequency are required to reduce the burden of frequent injections for patients. 

Davis developed an eye drop formulation using curcumin-loaded nanoparticles by TPGS/Pluronic F127, which enhances curcumin solubility by nearly 400,000 times [102]. This formulation remains stable in both liquid and lyophilized forms at room temperature for at least two months. Alarmable cell viability studies showed that incubating immortalized R28 cells in both the treatment and vehicle groups dramatically decreased glutamate-induced toxicity. The inclusion of α-tocopherol (TPGS) in the formulation may play a protective role against this toxicity, although TPGS alone did not protect against damage from cobalt chloride, suggesting potential additive effects of curcumin and TPGS. Previous research indicated that a 10 µL intravitreal injection of 0.5% (*w*/*v*) TPGS provided neuroprotection against ischemia/reperfusion injury in a rat model, an effect not observed with topical application, likely due to lower concentrations reaching the retina [30]. However, the current study shows that topical TPGS loaded with curcumin enhances RGC survival, possibly by modulating P-glycoprotein (P-gp) activity to enhance curcumin’s transocular transport [104]. There were no significant changes in IOP between the experimental and the OHT groups, indicating that the neuroprotective effects are independent of IOP changes [102].

Functional testing to distinguish between the effects of the carrier and the active drug made it possible to clarify their potential synergistic interactions and contribute to optimizing treatment strategies for eye diseases wherein IOP reduction alone is insufficient. Such tests could be crucial for understanding the mechanisms underlying the observed therapeutic benefits and refining approaches to glaucoma treatment.

### 2.6. Epoetin Beta (EPOβ)

Erythropoietin (Epo) is a glycoprotein hormone primarily known for stimulating erythropoiesis, which involves activating the bone marrow to produce red blood cells [105]. In addition to its hematopoietic role, Epo also has protective effects in the retina, such as resistance to inflammation, oxidative damage, ischemia, degeneration, and permeability problems [106]. It has been found to inhibit apoptosis in RGCs, maintaining visual function in various glaucoma models. Epo’s anti-apoptotic actions are linked to a variety of pathways, including caspase-3 downregulation via the PI3K/AKT pathway, mitochondrial cytochrome release inhibition, and calcium in the cell regulation [107]. Different methods of Epo administration to the retina, such as intravitreal, systemic, and subretinal, have been investigated. Different administration modalities may have varying risk profiles and side-effects. Systemic treatment could result in systemic adverse effects including increased hematopoiesis, hypertension, and thrombosis [108], whereas intravitreal injections may lead to ocular issues such cataract, vitritis, choroidoretinitis, retinal detachment, and endophthalmitis [79]. Table 2 summarizes the general advantages and disadvantages of various modalities for ocular drug delivery. To harness the therapeutic potential of Epo while minimizing these risks, Silva et al. developed a formulation binding with chitosan. This formulation is designed for the intraocular and topical administration of recombinant EPOβ, with the aim of improving retinal health through localized, targeted therapy, thereby reducing the likelihood of both systemic and severe local complications.

Sliva et al. designed a nanoparticle-based system for sub-conjunctival administration carrying EPOβ and conducted safety and release profile evaluations for both the carrier and the drug-loaded groups [109]. The cytotoxicity test cell lines confirmed the formulation’s safety, as no toxicity was observed. The study also monitored hematocrit levels before and after administering the EPOβ encapsulated in chitosan and hyaluronic acid, with results showing no significant changes, further affirming the safety of the formulation. ERG assessments performed pre- and post-injection demonstrated no detrimental effects on retinal health, indicating good biocompatibility of the vehicle group, regardless of whether they contained encapsulated EPOβ. In terms of drug release, lacrimal fluid simulation experiments showed that about 65% of EPOβ was released within the initial 16 min, with around 90% released over six hours. Immunofluorescence assays on ocular tissues revealed that the subconjunctivally administered drug-loaded group effectively transported EPOβ to the retina [109]. The detectability of EPOβ in the retina up to 21 days post-administration, with complete clearance by day 28, underscores the formulation’s capability to deliver and maintain therapeutic levels of EPOβ in the retina for an extended period.

Silva et al. conducted further studies about this formulation in a rat model topically [107]. They used ERG to measure retinal function at intervals of 3, 7, 14, and 21 days after inducing glaucoma. On day 21 under scotopic conditions, the treatment group demonstrated significantly higher b-wave amplitudes (198 ± 51 μV) compared to the control group (145 ± 39 μV), although the overall differences between the groups did not reach statistical significance. The treatment group showed a quicker recovery trend with both a-wave and b-wave amplitudes nearing baseline over time. Measurements of retinal thickness near the optic nerve indicated a marked improvement in the treatment group by day 21, with an average thickness of 145.6 μm versus 120.2 μm in the control group [107]. This reflects an improvement in retinal function. EPOβ presence was detected in the GCL, inner nuclear layer (INL), and outer nuclear layer (ONL) on days 7, 14, and 21. By day 14, EPOβ was found in the cornea and, by day 21, in the ciliary body and sclera, indicating that it infiltrated the cornea. The nanocarrier efficiently transported the active drug into the eyeball, as shown by the persistence of fluorescence signals 21 days after delivery. In addition, there was a significant difference in retinal thickness between the treatment group and the control group on day 21: 120.2 ± 10.6 µm and 145.6 ± 22 µm.

In the control group of the study, a decrease in IOP was noted following cauterization, which possibly aided in retinal recovery, with IOP normalizing by the seventh day post-operation. Previous studies suggest that temporary retinal damage caused by increased IOP in rats can fully reverse in three weeks [110]. To better mimic chronic conditions and extend the period for assessing the neuroprotective and neurodegenerative effects of the CS/HA-EPOβ nanoparticles, it might be beneficial to cauterize additional episcleral veins beyond the three used in this experiment. Furthermore, the experimental setup included only treatment and empty carrier groups, lacking a pure drug group for comparative analysis. Chitosan, known for its adhesive properties due to positively charged amino groups, enhances the corneal residence time of eye drops. Chitosan nanoparticles can bind to negatively charged sialic acid residues on the conjunctival and corneal mucosa, extending the retention time of the drug [111]. The absence of a pure drug group makes it challenging to determine whether the nanoparticle carrier indeed improves the drug’s bioavailability, targeting, stability, or other therapeutic effects.

### 2.7. N-Acetylcysteine (NAC)

NAC, an acetylated form of the amino acid cysteine, was first patented in 1960 and introduced to medicine in 1967 [112]. It is known for its mucolytic properties and acts as a powerful anti-inflammatory and antioxidant agent. NAC is used as a dietary supplement and has several medical applications [113]. It serves as a mucolytic agent in chronic pulmonary conditions and is frequently employed to manage paracetamol overdose [113]. It is also used to protect the kidneys in contrast-induced nephropathy and to prevent atrial fibrillation. NAC is also being investigated for its potential to modulate various pathophysiological processes in neurological and psychiatric disorders, acting on factors such as neuroinflammation, mitochondrial dysfunction, oxidative stress, apoptosis, and neurotransmitter systems involving glutamate and dopamine [114].

Pitha et al. present a PAMAM dendrimer (D-NAC) covalently coupled to the drug NAC, which lowers microglia activation, and RGC death following an individual salvage administration one week after laser therapy [115]. The carrier hydroxyl PAMAM dendrimer targeted activated retinal microglia/macrophages and was retained for up to about one month after administration. Compared to intravitreal injections, wherein D-Cy5 (PMAM dendritic polymer fluorescent marker) uptake was seen only in the retina, after intravenous injection, D-Cy5 was seen only in the optic nerve, and D-Cy5 was not present in the retina as a whole outside of the ONH [115]. This suggests that systemic administration of PAMAM dendrimer is unable to cross the outer retinal barrier. A single intravitreal dose of D-NAC given 1 week after IOP elevation significantly reduced the transcription of pro-inflammatory (IL-1β, IL-6, MCP-1) and A1 astrocyte (Serping1, Amigo2, Fkbp5) markers and increased retinal ganglion cell survival (39 ± 12%) compared to free NAC- (26 ± 14%) and BSS-treated eyes (20 ± 15%, *p* = 0.02) which had lower survival rates [115]. This highlights the neuroprotective effect of the combination of dendritic polymers with the antioxidant NAC and demonstrates the potential of PMAM dendritic polymers for early glaucoma detection and as a vehicle for neuroprotective therapy.

It is worth noting that this experiment did not include a vehicle-only group; had a vehicle group been established, it might have been more straightforward to observe whether there was a superimposed or antagonistic effect of NAC on dendritic combination. Both the treatment group and the drug-only group showed a decrease in IOP after administration, and it is challenging to determine whether the increase in RGC survival was attributable to the decrease in IOP. After 6 weeks, it might be a little late for enucleation, and it may be more appropriate to remove the lenses and perform immunostaining if the IOP has not returned to normal. In addition, incorporating functional in vivo testing could also strengthen efficacy evaluations, offering direct evidence of the treatment’s impact on visual function.

### 2.8. Melatonin

Melatonin is a neurohormone produced by the pineal gland and ocular structure [116]. It possesses significant antioxidant properties, lending itself well to potential therapeutic applications [116], with the demonstration that several of its metabolites help to scavenge free radicals. Melatonin modulates the activity of antioxidant enzymes such as superoxide dismutase (SOD), catalase, and glutathione peroxidase in the retina, which are enzymes protecting the retina from free-radical-induced damage [117]. In addition, melatonin effectively inhibits the nitric oxide pathway, affects glutamate uptake, and modulates the activity of key enzymes in the retina—increasing glutamine synthetase (GS) activity and decreasing glutaminase activity in golden hamsters [116]. These properties suggest that melatonin or its analogues could be effective in glaucoma treatment through the protection of retinal ganglion cells [118].

Dal et al. designed a topical formulation (MelAgo) combining melatonin with its analogue agomelatine, and compared it with two glaucoma medications, brimonidine and timolol [118]. They found that MelAgo had a better IOP-lowering effect than either timolol or brimonidine, and it was about twice as effective as either drug. Electrophysiological examination revealed that neither the vehicle nor timolol affected the Photopic Negative Response (PhNR) amplitude, with brimonidine, melatonin, and agomelatine almost restoring it to control level. The PERG test also showed that neither the vehicle nor timolol improved the amplitude or implicit time. However, brimonidine, melatonin, and agomelatine were able to restore the amplitude and implicit time. Conversely, RGC survival aligned with electrophysiological tests, indicating that RGC density nearly returned to normal levels after MelAgo treatment, but not completely following treatment with brimonidine, melatonin, or agomelatine. Although timolol and brinzolamide had comparable IOP-lowering effects, brimonidine demonstrated superior neuroprotective efficacy. Importantly, due to its potent antihypertensive effects, the formulation of melatonin and agomelatine effectively counteracted RGC loss and restored RGC function by inhibiting inflammation associated with gliosis [118]. Table 6 below summarizes the effects on IOP, retinal function, anti-inflammatory, and RGC density for MelAgo, timolol, and brimonidine for methylcellulose-treated eyes, as described by Dal et al.

However, it is plausible that the efficacy of MelAgo on ganglion cell survival corresponds to decreased eye pressure, albeit direct neuroprotection cannot be ruled out. This model involves elevated IOP, and it could be more effective to establish a glaucoma model with normal IOP to distinguish between direct neuroprotection and indirect neuroprotection resulting from a decrease in IOP. In addition, setting up a drug-only group (melatonin + agomelatine) might better demonstrate the effectiveness of the vehicle on drug bioavailability.

### 2.9. MicroRNA (miRNA)

MiRNAs are short non-coding RNA sequences that modulate gene expression post-transcriptionally, affecting processes such as apoptosis, cell growth, and differentiation [119]. Research indicates that miRNAs could be valuable therapeutic agents for diseases like glaucoma, with specific miRNAs targeted by synthetic analogs or inhibitors. miR-124, abundantly present in the central nervous system, encoded by three distinct genomic loci, named miR-124-1, miR-124-2, and miR-124-3, and has shown potential in early therapeutic tests [119,120]. It influences neuronal differentiation and has anti-inflammatory properties by promoting an M2 phenotype in microglial cells and macrophages, providing neuroprotection [121]. These properties are particularly beneficial [121] in reducing apoptosis in RGCs, crucial for combating the progressive degeneration seen in glaucoma.

A study delivered miRNA-124 and brinzolamide (BRZ) encapsulated in PEG–PSA nanoparticles to the retina via intravitreal injection, demonstrating good efficacy and neuroprotective effects [122]. Two animal models were used: a high IOP model and an optic nerve crush (ONC) model. In the high IOP model, the microbeads + miRNA/NP–BRZ group showed the most significant reduction in IOP, surpassing both the microbeads + PBS group and the microbeads-only group [122]. The ONC model was utilized to evaluate the neuroprotective efficacy of the formulation, and the results indicate that co-administration of miRNA/NP–BRZ significantly inhibited RGC apoptosis [122]. Both the fluorogold labeling method and TUNEL staining confirmed the significant reduction in RGC apoptosis with the combined miRNA/NP–BRZ treatment [122].

While it is well known that BRZ effectively lowers IOP [123], this study did not include a comparison group with BRZ + microbeads to determine whether the addition of miRNA affects the IOP-lowering efficacy. Additionally, the ONC model’s control group only included PBS, lacking a comparison with pure drug and pure carrier groups. Including these groups would have provided a more comprehensive assessment of the formulation’s potential and the synergistic effects of miRNA and BRZ.

## 3. Limitations

Despite the progress in the development of ocular drug delivery systems, a number of barriers still exist. Rodent models contribute much valuable data in glaucoma research, yet they fall short in replicating the specific anatomical features, progressive progression, and pathophysiology of the disease in humans. One major limitation is that rodents do not have a macula, a critical aspect when studying diseases like glaucoma that primarily affect human vision [124]. Additionally, the rapid progression of disease in these models, due to rodents’ shorter lifespans, does not accurately represent the typically slow progression of glaucoma in humans, wherein retinal and RGC damage unfolds over years rather than the weeks to months observed in rodents. There is a clear need for new animal models that more closely mirror the chronic neurodegenerative nature of human glaucoma [124]. 

Larger animals like rabbits and pigs might be more suitable owing to their longer lifespans and larger eye sizes, which are more comparable to humans. However, the use of larger animals is often limited by higher costs, greater maintenance requirements, and facility constraints. Rabbits, in particular, share key anatomical similarities with the human eye, such as the lamina cribrosa and astrocytes [125]. The composition of the aqueous humor in rabbits, which includes amino acids and free peptides, closely parallels that of humans. This makes the rabbit eye an excellent model for studying ocular biochemistry. Additionally, this animal model offers a straightforward, cost-effective, and reproducible option for preclinical eye disease research. Rabbits are gentle, have appropriately sized eyes for various techniques, and possess a relatively longer lifespan compared to other animal species [126].

However, rabbit models have limitations, including thinner scleral thickness, higher choroidal blood flow, and a lower vitreous volume, approximately 1.5 mL less than that of humans [127]. Additionally, rabbits have a larger and thicker lens that occupies more of the vitreous cavity [128], which may complicate the collection of samples from the posterior segment for biochemical analysis.

The retina of pigs and mini-pigs shares more similarities with the human retina than that of other mammals, including dogs, cats, goats, and cows [129]. In terms of ocular anatomy, pigs have a retina that closely resembles the human eye, featuring holangiotic retinal vasculature, a lack of tapetum, cone photoreceptors in the outer retina, similar scleral thickness, and three types of RGCs. These characteristics aid in studying the mechanisms behind selective RGC death [130]. Pigs are also considered easy to handle, grow slowly, and have appropriately sized eyes for diagnostic tools such as OCT, corneal topography imaging, and ERG compared to non-human primates. 

Additionally, given that glaucoma is a multifactorial disease, most existing models are insufficient as they typically only simulate one aspect of the disease’s progression. The variability among different animal models means that the effects of the same drug can differ markedly, making it essential to choose an animal model that aligns well with the drug’s mechanism of action. Furthermore, if a drug has an IOP-lowering effect, it is crucial to determine whether its neuroprotective actions are due to lowering IOP or a direct effect on neural tissues. This differentiation could be better understood by employing two models: one with elevated IOP and another with normal IOP, to isolate the effects of the drug on neuroprotection independent of IOP changes.

The current safety assessment of nanocarriers heavily relies on cell culture models due to their cost-effectiveness and simplicity [131]. However, these models are limited in terms of evaluating prolonged toxicological exposure as they do not sustain cell viability long enough. Additionally, nanocarriers can encounter stability issues during long-term storage, potentially skewing toxicity evaluation results. While there is no universally accepted method for assessing nanocarrier toxicity, factors such as surface charge, size, and solubility are considered potential indicators, yet extensive research is needed to validate these parameters. Most toxicity studies investigating nanocarriers have been conducted in animal models, with a significant gap in long-term clinical trials involving humans. Furthermore, for topically administered therapies, the frequent dosing required raises concerns about the accumulation of drugs and carriers, which could pose safety risks over time [131].

Some studies have used the RGC-5 line in cell culture. However, this is controversial as it is now known to be derived from mice rather than rats, and additional cytogenetic tests, karyotype analysis, as well as genetic and protein analyses, have revealed that these cells are not actually retinal RGCs but rather the 661 W cell line, which is a photoreceptor cell line transformed with the SV-40 T antigen from mice [132]. This indicates that RGC-5 cells are not a good representative model for RGCs. In contrast, the ARPE-19 cell line, derived from the human retinal pigment epithelium, should serve as a better alternative for studies involving retinal cells.

A key aim of neuroprotective treatments for glaucoma is to preserve RGCs during the initial phases of the disease, preventing their complete degeneration. Consequently, advanced techniques for the early diagnosis of glaucoma are essential. However, most in vivo experiments primarily focus on measuring IOP without conducting functional tests like ERG or VEP. Even when ERG is used, protocols that specifically isolate RGC responses from general retinal activity may be more appropriate than standard full-field ERG measurements of the a-wave, b-wave, and their implicit times [133]. For instance, pattern ERG (PERG) and PhNR, which is the negative wave after b-wave, could provide a better assessment of the RGC’s function [133]. Additionally, a novel technique called Detection of Apoptosing Retinal Cells (DARC) utilizes the fluorophore DY-776 conjugated to an Annexin A5 protein, termed ANX776. This molecule binds to apoptotic cells and can be imaged using confocal scanning laser ophthalmoscopy (cSLO). By monitoring changes in the number of labeled spots, the survival of RGCs can be assessed more accurately [134]. Implementing these advanced in vivo diagnostic and monitoring techniques can significantly enhance the evaluation of neuroprotective therapies’ effectiveness in preserving RGC function and viability in glaucoma research.

Finally, in studies exploring nanocarriers for delivering neuroprotective drugs, some experiments lack control groups with pure drugs or pure vehicles to compare with treatment groups. A comprehensive intergroup design, including these control groups, could better demonstrate the improvements that nanocarriers provide in therapeutic efficacy. This approach would offer clearer insights into the specific contributions of the nanocarriers and the drugs they deliver, thereby strengthening the validity and applicability of the findings.

## 4. Future Work

It is well-known that, while intravitreal injections can bypass the BRB and likely offer better efficacy, their invasive nature may lead to side effects such as infection, inflammation, and bleeding, thereby making non-invasive eye drop administration more attractive. Combining nanocarriers can overcome the issues of poor bioavailability and limited delivery to the retina associated with topical administration. However, current studies using eye drops often require very high administration frequencies, nearly 1–2 times daily, raising concerns about drug accumulation, as previously mentioned. Eye drops incorporating chitosan offer potential advantages, as chitosan can relax tight junctions on the corneal epithelium, provide mucoadhesiveness due to its positive charge, and extend drug release time [135]. These benefits suggest a reduced administration frequency and improved safety for topical delivery. However, the safety of chitosan nanoparticles themselves has not been extensively studied in clinical research, necessitating further clinical research to confirm their safety and efficacy. Future studies should also try to elucidate the optimal physicochemical properties of chitosan for delivering glaucoma drugs, such as the best zeta potential, amination level, molecular weight, and degree of deacetylation.

Another non-invasive delivery method with potential is intranasal administration. Khan et al. demonstrated that, after intranasal administration, drugs rapidly accumulate in the eyes and optic nerves of rodents within 30 min, likely by directly entering the central nervous system and traveling along the optic nerve to the retina, thus bypassing the blood–brain barrier (BBB) and the BRB [136]. This method offers the advantages of low dosing and reduced systemic risk compared to oral administration [137]. Comparing the efficacy of the same formulation via eye drops and intranasal delivery could be a novel direction for glaucoma treatment, addressing the issue of poor patient compliance with simple administration methods.

The complicated pathophysiology of glaucoma comprises several routes and processes. To prevent RGC apoptosis and delay disease progression, targeting a specific pathway or cause may not be sufficient. Multi-drug therapies, which simultaneously target multiple pathways and mechanisms or regulate key modulators affecting multiple downstream effectors, offer a promising approach to neuroprotection and could be more effective. Delivering multiple drug formulations with different physicochemical properties using conventional solvents (e.g., PBS and glucose solutions) is challenging, but feasible with nanocarriers. For example, studies on ONC mouse models have shown that combined AAV2 gene therapy for BDNF and TrkB provides more sustained and effective results than either therapy alone [138]. Topical administration of melatonin/agomelatine nanomicelles significantly reduces elevated IOP, and the combined use of both melatonin compounds prolongs the hypotensive effect compared to using either drug alone [139]. Understanding innate compensatory mechanisms is critical to designing combination medicines. As a result, personalized multi-drug therapies based on nanocarriers adapted to individual patient physiology could become the new mainstream treatment. This may involve developing novel nanocarriers that simultaneously deliver multiple active drugs targeting different mechanisms or distinct pathways within the same mechanism to delay RGC apoptosis. By combining multifunctionality and targeted delivery, these nanocarriers could address multiple pathways involved in RGC degeneration, providing a more comprehensive neuroprotective strategy for retinal diseases.

The majority of novel drug delivery techniques were predominantly tested in vitro studies or on animals, thereby lacking detailed in vivo experimentation in human eyes. Their systemic and metabolic side effects remain unclear. Current experiments remain in the preclinical stage; therefore, further clinical translational studies are required due to the anatomical differences between animal models and human eyes. More research is required to fully understand the safety and repeatability of combining carriers with neuroprotective drugs. Large-scale, long-term clinical studies are anticipated. However, transitioning these nanotechnology-based drug delivery systems from the experimental stage to practical clinical use faces significant challenges, particularly in scaling up production and maintaining consistent quality control scale. Additionally, the impact of different intraocular environments, including history of eye surgery, repeated implants, or vitrectomy patient, on various characteristics of nanocarriers also warrants investigation [10]. For instance, nanoparticle movement post-operation in cataract or vitrectomy with silicone oil [140], which animal models cannot simulate, are important considerations. Thus, surgical history is a significant factor that merits attention in further clinic studies.

In the future, greater emphasis should be placed on developing novel non-invasive ocular drug delivery systems that can effectively overcome ocular barriers, extend drug release duration, and maintain therapeutic concentrations at the targeted lesion sites. To achieve this, optimizing key properties of nanocarriers—such as size, zeta potential, refractive index, safety, stability, pH, surface tension, and osmotic pressure—is crucial. Additionally, more extensive in vitro and in vivo studies are needed, alongside the development of animal models that more closely mimic human eye diseases.

## 5. Conclusions

Glaucoma is a vision-threatening disease affecting patients of all ages globally. While current treatments primarily focus on lowering IOP to slow disease progression, they are not curative. Neuroprotective therapies are gaining increasing attention, as they aim to directly prevent or delay the apoptosis of irreplaceable neuronal cells, particularly RGCs. Such treatments can be used alone or in conjunction with IOP-lowering therapies to achieve complementary or synergistic effects and better therapeutic outcomes. As research into neuroprotective therapies advances, it enhances our understanding of the mechanisms underlying glaucoma onset and progression. This knowledge could eventually lead to preventative strategies and potentially the restoration of neural cell function, greatly benefiting both healthcare systems and patients’ quality of life. Nanocarriers loaded with neuroprotective agents can address several limitations, such as poor penetration of the BRB, suboptimal pharmacokinetics, and short half-life in vivo. By improving drug bioavailability, targeted delivery, and efficacy, nanocarriers can reduce the required drug dosage, thereby minimizing adverse reactions, provided the carriers themselves are safe. Additionally, nanocarriers can simultaneously deliver both hydrophilic and hydrophobic drugs by encapsulating them within cavities or by using covalent bonds to bind the drugs. This capability allows for a single formulation to carry multiple active drugs, facilitating combination therapy. Although preclinical investigations have shown positive results, few neuroprotective nanocarrier treatments have made it into clinical trials. More clinical research is required to confirm their therapeutic effects. The path to effective neuroprotection in glaucoma remains long, but neuroprotective nanocarriers have demonstrated great potential toward becoming the next generation of mainstream treatments for glaucoma.

## Figures and Tables

**Table 4:** Efficacy and safety of different nanocarrier formulations of brimonidine.

Neuroprotective Drug	Nanocarrier	Efficacy	Safety	Key Findings
Brimonidine	Standard eye drops–Topical concentrations: 0.1%, 0.15%, 0.2%	Limited bioavailability (1–7%) and rapid clearance necessitate frequent dosing.	Common side effects include asthenia; drowsiness; eye discomfort (dry eye, eye inflammation); hyperemia; hypersensitivity; sensation of foreign body; taste altered.	Limited by frequent administration needs due to low bioavailability.
	Polydopamine (PDA)	Enhances RGC protection and significantly boosts axon regeneration compared to brimonidine alone; superior efficacy in reducing microglial activation and increasing visual function.	Demonstrates good ocular biocompatibility; intravitreal injections show no adverse effects.	PDA nanoparticles provide a synergistic neuroprotective effect, improve RGC density, and boost visual outcomes.
	Brimonidine with human serum albumin (HSA) nanoparticles	Prolongs RGC survival, extending treatment efficacy compared to brimonidine alone.	High biocompatibility: no toxic effects reported.	HSA improves brimonidine’s duration of action, suggesting potential for more sustained neuroprotection.
	alkoxylphenacyl-based polycarbonates copolymerized with polycaprolactone (AP-PCL)	Sustained release up to 90 days with consistent therapeutic levels; effective long-term IOP management.	Well tolerated; no retinal detachment observed in vivo; no toxic effects on human trabecular meshwork cells.	Demonstrates potential for extended-release formulations, reducing dosing frequency.
	Brimonidine with nanosponges	Effective IOP reduction sustained for up to 3 weeks, depending on nanoparticle size; promising for long-term management.	No major adverse effects reported in animal studies.	Enhanced IOP control with longer-lasting effects compared to standard formulations, though further testing is needed for functional RGC protection assessment.

**Table 5:** Efficacy and safety of different nanocarrier formulations of brain-derived neurotrophic factor (BDNF).

Neuroprotective Drug	Formulation/Approach	Efficacy	Safety	Key Findings	References
**Brain-Derived Neurotrophic Factor (BDNF)**	**BDNF (Standard Injections)**	Provides neuroprotection to RGCs by acting directly through TrkB receptors or indirectly via glial cells. Effective in improving RGC survival.	Generally safe, but repeated injections are needed due to the transient nature of BDNF, which can raise safety concerns over time.	Challenges include rapid degradation, limited nuclear translocation, and difficulty sustaining therapeutic levels.	[68]
	**K2^®^ Nanoparticle-Based BDNF Gene Delivery**	Superior retinal transfection and increased gene expression levels compared to conventional methods; 3.4× higher BDNF levels in animal models.	Good biocompatibility: no major adverse reactions noted in studies.	Effective BDNF gene delivery via nanoparticles demonstrated in co-culture models; promising for sustained gene therapy.	[70]
	**Combined BDNF and TrkB Gene Therapy**	Provides superior neuroprotection and axonal transport compared to individual therapies; prevents receptor downregulation, ensuring sustained activation.	Safe in experimental settings.	Synergistic effects improve therapeutic outcomes, offering enhanced and prolonged RGC protection.	[72]
	**PACAP38-Linked Exosomes (Non-Glaucoma Model)**	Markedly improves survival rates, nerve layer thickness, and visual recovery in traumatic optic neuropathy models.	High biocompatibility observed; no significant adverse effects reported.	Demonstrates the effectiveness of neurotrophic factor delivery via exosomes for enhanced neuroprotection.	[73]

**Table 6:** Summarizing findings of the effects of MelAgo, timolol, and brimonidine on intraocular pressure (IOP) and retinal ganglion cell (RGC) survival.

Agents	IOP Reduction	Photopic Negative Response	Pattern ERG	Gliosis-Related Inflammation	RGC Density (% of Healthy Control)
MelAgo	60%	PhNR amplitude close to normal.	Reduced MCE-induced prolonged implicit time.	Reduced Iba1 and GFAP by 2.3- and 3.2-fold. Reduced levels of TNF-α, IL-1β and IL-6 (by 2.0-, 2.1-, and 2.3-fold). Increased levels of IL-4 and IL-10 (by about 2.0- and 2.5-fold).	Central 92 ± 5 Middle 94 ± 5 Peripheral 94 ± 5
Timolol	32%	No effects.	No effects.	No effects.	Central 78 ± 4 Middle 83 ± 3 Peripheral 81 ± 4
Brimonidine	34%	PhNR amplitude close to normal.	Reduced MCE-induced prolonged implicit time.	Reduced GFAP by 1.6-fold.	Central 88 ± 3 Middle 91 ± 6 Peripheral 91 ± 3
